# Terminal Ileitis due to *Yersinia* Infection: An Underdiagnosed Situation

**DOI:** 10.1155/2020/1240626

**Published:** 2020-05-23

**Authors:** John K. Triantafillidis, Thomas Thomaidis, Apostolos Papalois

**Affiliations:** ^1^Metropolitan General Hospital, Athens, Greece; ^2^Universitätsmedizin Mainz, Germany, “Hygeia” Hospital, Athens, Greece; ^3^Experimental, Educational, and Research Center ELPEN, Athens, Greece; ^4^European University Cyprus, School of Medicine, Nicosia, Cyprus

## Abstract

Endoscopy is currently the gold standard for the diagnosis of inflammatory bowel disease (IBD). The presence of macroscopic lesions along with the microscopic detection of inflammatory infiltration in the terminal ileum often leads the gastroenterologist to the diagnosis of Crohn's disease (CD). However, some of these cases could be, in fact, an infection caused by *Yersinia* spp., accompanied or not with CD, which could be easily diagnosed with the identification of serum antibodies against *Yersinia* outer protein antigens (YOP antigens). Since Yersiniosis is considered to be an uncommon situation, food and water are not usually checked for the possibility of contamination by *Yersinia*. Therefore, it is reasonable to assume that the true prevalence of *Yersinia* infection in patients with terminal ileitis is probably underestimated. In this article, we review the most important data regarding the various aspects of *Yersinia* infection with special focus on its pathophysiology and diagnosis. We recommend testing for serum antibodies against YOP antigens in all patients with an endoscopic and histological image of terminal ileitis in order to identify Yersiniosis in conjunction or not with terminal ileum CD.

## 1. Introduction

The genus *Yersinia* (Y.), the causative agent of Yersiniosis in humans, includes three different Gram-negative coccobacilli species, namely, *Y. enterocolitica* (YE), *Y. pseudotuberculosis* (YP), and *Y. pestis*, the latter being the causative agent of the bubonic and pneumonic plagues. Among the three types of Yersiniosis, YP isolates are pathogenic for human, whereas YE strains include pathotypes of different virulence [1]. Generally, *Yersinia* strains are psychrotrophic bacteria that are resistant to many environmental factors.

During the last years, inflammatory lesions in the terminal ileum mucosa are increasingly recognized due to the easy endoscopic access. Moreover, the histological detection of inflammation in symptomatic individuals has prompted the endoscopists to hastily diagnose Crohn's disease (CD), in the absence of recent history of drug consumption or viral infection. However, a number of these cases could in fact correspond to *Yersinia* infection, as tests for the presence of serum antibodies against *Yersinia* outer protein (YOP) antigens are usually not performed. Therefore, it is reasonable to assume that some cases characterized as CD, especially those of mild severity, are in fact cases of Yersiniosis that resolve spontaneously or are followed by a treatment with ciprofloxacin, an antibiotic commonly used by most gastroenterologists worldwide in CD patients. It is unknown if the coexistence of *Yersinia* infection and CD in the same patient increases the severity of the underlying enteropathy. Also, the ability of *Yersinia* to survive in natural samples and thrive at refrigeration temperatures means that the true contribution of this pathogen to disease might be underestimated.

The aim of this review is to summarize the most important data regarding the various aspects of *Yersinia* infection in humans, with special emphasis on its pathophysiology and diagnosis. We also reviewed the data referring to the possibility of *Yersinia* infection in patients diagnosed with terminal ileitis (TI) and emphasized the available diagnostic tools, especially the serum estimation of antibodies against YOP antigens.

## 2. Epidemiological Aspects of *Yersinia* Infection

YE has been isolated from patients in many parts of the world, but it appears to occur predominantly in cooler climates, including northern Europe, Scandinavia, and Japan. The prevalence of infection is higher from November to January. In the USA, YE infection accounts for 5% of enteric infections among children younger than 5 years old. The isolation of YE in developing countries is uncommon [2]. YE is transmitted to humans via water, food, soil, and animals. The most important reservoirs are rodents, domestic animals, and birds. Pork products, as well as minced meat, can be contaminated with insects being spread to other meat cuts during slaughter. In a herd, *Yersinia* can spread from one pig to another. YE has also been isolated from flies found in farm piggeries and kitchens suggesting that arthropod vectors/insects could contribute to the transmission of *Yersinia* from animals to humans [3].

The infection is transferred predominantly through the faecal-oral route. Pork consumption (especially undercooked) or raw pork products are responsible for Yersiniosis. Outbreaks have also been reported from drinking water contaminated with this pathogen. There are case reports of infection being transferred from an infected household pet and via transfused blood products. It is important to emphasize that infected individuals may shed YE in stools for at least 90 days after the symptom resolution.

Dairy products, including cheese, are generally thought to be safe, although there is a possibility of contamination during or after the production process. Nevertheless, YE is the third most common enteric pathogen responsible for food poisonings with dairy products. Zadernowska et al. showed that blue cheese may represent a suitable environment for YE growth. Bearing in mind the fact that YE can grow under refrigerating conditions, they may present a real threat to human health [4].

In patients diagnosed with TI, an infectious cause could be found in one-third of the cases; including *Yersinia* spp., CD could also be demonstrated in up to 12.1% of patients [5]. Taking into account these two most important conditions, namely, *Yersinia* infection and CD, we can conclude that TI could be manifested under three conditions: TI due to *Yersinia* infection, TI due to CD, and TI due to the coexistence of *Yersinia* infection with CD. *Yersinia* species are frequently found at low levels in the terminal ileum of both healthy individuals and patients with TI. According to recent data, Y. infection was no more likely to be detected in CD tissues than those from inflammatory and noninflammatory controls [6]. However, in a study aimed at determining the seroprevalence of anti-*Yersinia* antibodies in 750 healthy Austrians using the recomBlot *Yersinia* western blot kit it, was found that the overall seroprevalence was 29.7%. Seroprevalence increased significantly with age, from 24.7% in the group of persons aged between 19 and 24 to 38.5% in the group of persons older than 44 years old. This high seroprevalence contrasts to the small number of cases reported suggesting the subclinical or mild nature of the infection [7].

Knösel et al. showed that, although several potential pathogens can be detected on tissue specimens from patients with CD, these pathogens could also be detected in control patients, suggesting that many infectious pathogens could be associated with CD, but they do not represent an obligatory cause [8]. In a follow-up study of 44 patients with CD tested for *Yersinia* infection, a significant proportion of patients (39%) were positive [9]. Finally, in a study from Germany, it was found that the mean annual incidence of Yersiniosis was 7.2/100,000 population and the higher incidence was found in children under the age of five. About 90% of infections were acquired domestically. The predominant serotype was O:3 [10].

## 3. Pathophysiology of Yersiniosis

### 3.1. Invasion

YE is an invasive organism that can cause disease by tissue destruction. The pathogen passes into the stomach via contaminated food and invades the epithelial cells of the small intestine and the underlying Peyer patches with the help of the so-called M cells (antigen-sampling intestinal epithelial cells). The epithelium overlying the Peyer patches has a high concentration of M cells. *Yersinia* produces three invasion proteins, Ail, YadA, and invasion. The latter has the ability of binding the *β*1-chain integrin receptors found at high levels on the luminal side of M cells. YOPs are associated with bacterial resistance to opsonization and neutrophil phagocytosis. The lymphatic link between Peyer patches and mesenteric lymph nodes results in bacterial dissemination [11]. It is of interest that *Yersinia* also secretes urease that breaks down blood urea in ammonia, which explains why *Yersinia* survives in the hostile gastric environment the same way that *Helicobacter pylori* does. It can also produce substances that can offer resistance to complement mediated opsonization and phagocytosis. The YOPs could arrest phagocytosis by blocking secretion of cytokines, including TNF-*α* and IL-8. The dissemination of the pathogen to extraintestinal sites is mediated via colonization of the Peyer patches resulting in the appearance of Yersinia in other tissues or bypassing the Peyer patches by going straight to the systemic circulation. The pathogenesis of reactive arthritis is likely due to an immune response to *Yersinia* antigens that crossreact with host antigens. The host may be positive for HLA-B27.

### 3.2. Enterotoxicity

The enterotoxin produced by YE plays a rather minor role in causing disease, as diarrhea could appear in the absence of enterotoxin. It is of interest that the toxin cannot be produced at temperatures higher than 30°C.

### 3.3. Iron and *Yersinia* Infection

The relationship between microbial infection, including *Yersinia* infection, and iron status received much attention during the last years. A large number of trials of iron administration in African and Asian children some years ago confirmed the occurrence of serious adverse events. It was reported that malaria, respiratory infections, and severe diarrhea, as well as various febrile situations of unknown origin could be observed during prolonged iron administration, although the underlying mechanisms are largely unknown [12–15]. Cross et al. investigated the *ex vivo* growth characteristics of exemplar sentinel bacteria [including Gram (-) (YE) and (+) bacteria] in adult sera before and after 4 hours of oral supplementation with 2 mg/kg ferrous sulfate. A bacterial overgrowth after iron supplementation was observed and was significantly correlated with transferrin saturation, suggesting that small doses orally administered could promote bacteremia by accelerating early phase bacterial growth [16].

It is well known that *Yersinia* has the unique property not to chelate iron, due to its inability to produce siderophores, a chelator absolutely necessary for this process. However, *Yersinia* can utilize siderophores produced by other bacteria. In the gastrointestinal tract, there is an abundance of siderophore compounds from other organisms. It should be emphasized that *Yersinia* is ferrophilic and capable of growth in stored units of red cells at cold temperatures. The increased pathogenicity of YE after iron overloading explains in part the varying degrees of virulence among YE serotypes. Many clinical reports confirmed the ability of *Yersinia* to cause serious clinical situations after iron overloading. Delicou et al. reported the case of a 43-year-old woman with a history of thalassemia and heavy iron overload, who presented with lower abdominal pain, maculopapular rash, and arthritis. Serological examinations revealed positive IgA and IgG antibodies against YopD(4a) and Yop M(2a) and IgG for LorV (V antigen). The patient was successfully treated with oral ciprofloxacin. Twelve months after the infection, only IgG antibodies remained positive [17]. Host defense mechanisms that aim to withhold iron from invading pathogens include innate strategies against infection [18]. A number of experimental studies confirmed that oral iron adversely modifies the gut microbiome and increases the virulence of enteric bacteria [19]. It is of interest that certain *Yersinia* strains produce an iron-binding agent that allows *Yersinia* to grow in a depleted iron state [20].

### 3.4. *Yersinia* Infection and Probiotics

The World Health Organization defines probiotics as “live microorganisms that, when consumed in adequate amounts as part of food, confer a health benefit on the host.” Probiotics include species of *Lactobacillus* and *Bifidobacterium* that are Gram-positive, lactic acid-producing bacteria found in the GI tract. Moreover, some dietary supplements may contain strains of *Enterococcus*, *Streptococcus*, and *Escherichia*, agents that are less commonly found in the intestinal tract [21].

The dose of probiotics varies depending on the different strains of bacteria and the underlying health condition. However, there is no recommended daily dosage for probiotics. Generally, probiotics are sensitive to environmental conditions, such as heat, moisture, oxygen, and light. Also, some—mainly theoretical—adverse effects include the potential for transmigration, colonization, or transfer of antibiotic resistance in immunocompromised patients, young children, and elderly patients in whom caution should be paid while prescribing probiotics. The mechanisms of action of probiotics are probably related, among many others, to the alteration of the composition of intestinal flora, intestinal mucosa immune reactions, and the stimulation of intestinal lactase activity.

An increased resistance to infection is one of the beneficial effects attributed to probiotics. This effect may be due to several mechanisms: production of inhibitory substances, blocking of adhesion sites on the intestinal surface, competition for nutrients, and stimulation of mucosal and systemic immunity.

During the last years, some data concerning the role of probiotics in *Yersinia* infection emerged. These data are summarized below.

#### 3.4.1. Aeromonas sobria GC2 and Bacillus subtilis JB-1

Subcellular components of the probiotics Aeromonas sobria GC2 and Bacillus subtilis JB-1, when administered to rainbow trout Oncorhynchus mykiss conferred protection against a new biogroup of *Y. ruckeri*. These results point to the potential of using cellular components of probiotics for the protection of fish against bacterial diseases [22].

#### 3.4.2. Bifidobacterium adolescentis

*B. adolescentis*-fed and *Yersinia*-infected mice were protected from *Yersinia* infection as indicated by a significantly reduced weight loss and splenic *Yersinia* load, when compared to *Yersinia-*infected mice. This was associated with increased intestinal plasmacytoid dendritic cell and regulatory T-cell frequencies, which might account for the B. adolescentis-mediated protection from YE infection [23].

#### 3.4.3. Enterococcus

In a relevant study, all the *Enterococcus* isolates from different dairy products exhibited inhibitory activity against several food spoilage bacteria, including YE, thus being candidates for the production of functional foods [24].

#### 3.4.4. Lactobacilli

Damodharan et al., in a study aimed at characterizing the antagonistic activity against YE of paracasei strain KNI9, found that this strain exhibited tolerance to simulated orogastrointestinal condition and antimicrobial activity against YE. They also found that the strain KNI9 inhibited the adherence and invasiveness of YE to the Caco-2 cell line indicating that this strain should be further developed into a potential probiotic strain having action against YE [25].

In another study, strains of *Lactobacillus* spp. were screened for their antagonistic activity in coculture against the ureolytic pathogen YE. A reduction in the pathogen population was observed after 6 hours when each *Lactobacillus* strain was cocultured with the pathogen causing an almost complete inhibition of urease activity [26].

Bujalance et al. have shown that twenty *Lactobacillus* strains were able to inhibit YE. This inhibition was mainly attributable to a decrease in pH resulting from dextrose fermentation by lactobacilli [27].

Six strains, including *Lactobacillus fermentum* (4 strains), *Lactobacillus paracasei*, and *Lactobacillus plantarum*, showed a good probiotic potential and inhibited the growth of enteropathogenic bacteria including YE ATCC 23715 [28].

The exposure of human epithelial cells to L. fermentum does not induce NF-*κ*B activation and subsequent IL-8 secretion in HeLa cells, whereas YE induces NF-*κ*B activation and high levels of IL-8. *L. fermentum* inhibits the YE-induced IL-8 production. Therefore, *L. fermentum* may have probiotic properties modulating intestinal inflammatory responses [29].

Lactobacillus plantarum C4 was tested for its protective and immunomodulatory capacity in a murine model of Yersiniosis. The inoculation of mice with a low pathogenicity serotype O9 strain of YE resulted in a prolonged intestinal infection with colonization of Peyer patches. The pretreatment with C4 shortened the colonization of Peyer patches. This protective effect was associated with a proinflammatory status in the intestinal mucosa (TNF-*α* production in infected mice was increased by C4) and an increase in total IgA secretion. Therefore, *L. plantarum* C4 can increase resistance to intestinal infections through its immunomodulatory activity [30]. The application of *Lactobacillus plantarum* (2 × 10^7^ CFU g^−1^ feed) on vaccinated rainbow trout (29.5 ± 2 g) against Yersiniosis was able to enhance the efficacy of immersion vaccination to *Yersinia ruckeri* [31]. Metabolites of the probiotic lactobacilli strain *L plantarum* 8P-A3 jugulate the development of pseudotuberculosis at an early stage of the pathological process in experimental animals infected with pathogen *Y. pseudotuberculosis* causing the preservation of the colonization resistance of the intestinal mucosa that prevents the adhesion and colonization of the pathogen [32].

#### 3.4.5. E. coli

Bosák et al. showed that 4 E. coli strains (EcH22, EcColinfant, Ec1127, and Ec1145), producing the antibacterial agent colicin F_Y_, were able to inhibit the growth of YE during cocultivation *in vitro*. In dysbiotic mice treated with streptomycin, E. coli strains producing colicin F_Y_ inhibited the progression of YE infections, suggesting that the antimicrobial activity of colicin F_Y_ may be used in the treatment of YE infections [33]. The strain H22 was shown to produce several antimicrobial compounds with inhibitory capabilities against pathogenic or potentially pathogenic enterobacteria [34]. The invasion of YE was inhibited by the probiotic E. coli strain Nissle 1917 [35].

An interesting study showed that the probiotic E. coli strain Nissle 1917 (EcN) seems to be of therapeutic clinical benefit in collagenous colitis. This may be explained with the antagonistic effect of EcN against *Yersinia* species, since a relevant number of patients suffering from collagenous colitis showed positive titres of serum IgG and IgA antibodies against *Yersinia* species [36].

#### 3.4.6. S. salivarius and B. adolescentis

*S. salivarius* inhibited the YE-induced NF-*κ*B activation in mice orally infected with YE and in mice with mucosae impaired by DSS treatment. *B. adolescentis*-fed mice also had a significantly lower mean pathogen burden in the visceral organs, intestinal TNF-*α* mRNA expression, and loss of body weight upon oral infection with YΕ [37].

#### 3.4.7. Enterobacter cloacae and B. mojavensis

The concomitant use of *Enterobacter cloacae* and *B. mojavensis*, as a feed supplement, can protect rainbow trout from Yersiniosis and limit the use of antibiotics in controlling the disease [38].

#### 3.4.8. Reactive Arthritis and Probiotics

Bacterial gut infections, including YE, may induce reactive peripheral arthritis, and 20% of these patients may develop chronic spondyloarthropathy. Patients with early arthritis could be managed with probiotics, prebiotics, and synbiotics, thus improving the quality of life and positively influencing the natural course of the disease [39].

### 3.5. Gastric Hypochlorhydria

Mice that were constitutively hypochlorhydric due to a mutation in a gastric H(+)/K(+)-ATPase gene were infected with YE. Significantly greater numbers of *Yersinia* survived in hypochlorhydric mice, resulting in reduced median infectious doses [40].

In summary, the abovementioned data suggest that certain probiotic strains might be promising beneficial agents in patients with gastrointestinal infections, including Yersiniosis. Future studies are necessary in order to accurately define the dose and duration of administration.

## 4. Clinical Manifestations

Intestinal Yersiniosis could be manifested as TI, enteritis, mesenteric lymphadenitis, pseudoappendicitis, and septicemia. The incubation period is typically 4 to 6 days (range 1 to 14 days). Acute infection may result to mucosal ulceration (usually in the terminal ileum and rarely in the ascending colon), necrotic lesions in Peyer patches, and mesenteric lymph node enlargement. Symptoms include diarrhea or bloody stools, abdominal pain, and fever. Infections with either YE or YP can be asymptomatic, mild, or severe, depending on the immune status of the host. The duration of diarrhea in acute Yersiniosis can range from 12 to 22 days. The infection usually resolves within a few weeks, with or without the use of antibiotics. However, complications, such as reactive arthritis, can manifest 1-4 weeks postinfection, with an increased risk if the individual is positive for the MHC HLA-B27 allele. YP is the least pathogenic of the *Yersinia* species and causes a zoonotic disease with symptoms similar to those of YE. Following the acute infection, the bacteria may continue to shed in stool for a median of 40 days (range 17 to 116 days). In most cases, the duration of symptoms before diagnosis varies from 1 to 2 weeks, while in a small proportion, the symptoms could be present for months before diagnosis [41].

Yersiniosis is difficult to distinguish from other causes of acute diarrhea. The localization of pain to the right lower quadrant may be diagnostic for Yersiniosis. Sepsis has been described in patients who are immunocompromised or in an iron overload state. Acute Yersiniosis can also mimic appendicitis (pseudoappendicitis) presented with right lower quadrant abdominal pain, fever, vomiting, elevated white blood count, and diarrhea. Emergency surgery demonstrates inflammation of the terminal ileum and mesenteric lymph nodes with a normal appendix [42].

In a study aimed at elucidating the long-term prognosis of patients with TI, it was found that isolated acute TI discovered on diagnostic ileocolonoscopy rarely leads to final diagnosis of CD (4.6%) and that only the presence of stricture on cross-sectional imaging may predict CD development [43]. In patients with *Yersinia* infection and symptoms suggesting acute appendicitis, MRI of the abdomen could show evidence of TI with a normal appendix. Further work-up in these cases might detect *Yersinia* infection [44]. *Yersinia* infection could also present with liver or spleen abscesses [45], bacteremia, septic arthritis [46], or cutaneous aseptic abscesses [47].

*Yersinia* infection could precede the diagnosis of CD. Zippi et al. described a patient with mesenteric adenitis due to YP who subsequently was diagnosed with CD [48]. At the moment, it is unknown whether the presence of the microorganisms is an epiphenomenon or actually a contributing factor in the pathogenesis of CD. Homewood et al. [49] described another case of TI due to YP infection. The patient was subsequently diagnosed with CD. A number of authors speculated that YP ileitis modified the classical features of YP by the preexisting CD [50–52]. Furthermore, YE DNA has been detected in the histology of colonic resections and mesenteric lymph nodes in a series of CD cases. In a relevant study, it was found that the incidence of inflammatory bowel disease was higher in patients with positive serum antibodies against YE than in the antibody negative group [53].

The incidence of reactive arthritis following YE infection varies in different countries. The most commonly affected joints are the knees and ankles. In the majority of cases, two to four joints become involved sequentially and asymmetrically over a period of a few days to two weeks. In two-thirds of cases, the acute arthritis persists for one to four months. Chronic joint disease or ankylosing spondylitis occurs rarely. Complications from the digestive system include bowel perforation, peritonitis, ulcerating ileitis and colitis, intussusceptions, paralytic ileus, cholangitis, mesenteric vein thrombosis, toxic megacolon, hepatic and splenic abscesses, liver failure, and small bowel necrosis, while extraintestinal complications include septicemia, renal abscess, osteomyelitis, lung abscess, endocarditis, suppurative lymphadenitis, skin infection, mycotic aneurysm, myocarditis, and glomerulonephritis.

Reinfection of *Yersinia* is possible and asymptomatic carriage may occur. Death is uncommon, but patients with comorbidities are at risk for YE bacteremia having a high fatality rate. Erythema nodosum represents another postinfectious sequel.

## 5. Diagnostic Methodology

Diagnosis depends on a detailed history, physical examination, and laboratory and imaging findings. Diagnosis may also be succeeded from positive cultures obtained from mesenteric lymph nodes, pharyngeal exudates, peritoneal fluid, or blood. Polymerase chain reaction and immunofluorescence assays have been developed. Endoscopic and imaging studies (ultrasound or CT scan) are often required in order to determine if the patient has appendicitis or pseudoappendicitis. Serologic tests, including ELISA assays and immunoblotting to detect IgG, IgA and IgM, are used in many countries. The detection of antibodies against YOPs has significantly contributed to our diagnostic armamentarium. Antibodies against the microorganism develop soon after infection and persist for a long period of time. Antibody levels begin to rise within the first week of illness, reach a peak level in the second week, and then return to normal within 3-6 months. However, antibodies may remain detectable for several years. IgA antibodies have been shown to persist for 14 to 16 months following the onset of infection. This contrasts with the persistence of IgA antibodies for 5 months in cases of Yersiniosis without complications. In cases of chronic enteritis, IgA antibodies against YOPE (23 kDa), YOPD (35 kDa), and YOPB (41 kDa) are being developed. IgA antibodies against YOPD develop in 90% of reactive arthritis cases. IgG antibodies develop more frequently against YOPE (23 kDa), YOPB (41 kDa), YOPD (35 kDa), and YOPH (51 kDa). They can persist longer in cases of reactive arthritis, but not as consistently as IgA antibodies. IgM antibodies persist for only 1 to 3 months following the onset of infection and are not as useful for the diagnosis of reactive arthritis. Western blot analysis utilizes YOPs for the detection of *Yersinia* antibodies against YE and YP, being significantly more sensitive and specific than the current complement fixation method. It also offers a differentiation between IgG and IgA antibodies, which may indicate the state of the disease. IgA and IgM antibodies appeared as acute-phase markers rapidly decreasing in the reconvalescent phase [54]. The existence of only IgG antibodies in the serum is indicative of past infection. Concurrent presence of both IgG and IgA antibodies indicates an ongoing infection [55].

However, when interpreting the absence of IgA antibodies, we must bear in mind the fact that a small proportion of the normal population does not produce IgA (selective IgA deficiency) [56]. Yersiniosis has also been described in a patient with selective IgM deficiency. In this patient, the western blot analysis revealed the presence of 5 positive IgG and 3 positive IgA serum YOP antigens [57]. The western blot assays allow the differentiation of specific antibody isotypes, including the specific detection of IgA antibodies, which are the most important for the diagnosis of reactive arthritis. It is useful as an aid in the diagnosis and management of enteric infection and might be a valuable tool in identifying *Yersinia* as a causative organism in reactive arthritis cases. In recent years, crossreactivity between Y., *Bartonella henselae*, *Borrelia burgdorferi*, *Brucella*, *Chlamydia pneumoniae*, and *Rickettsia rickettsii* species has been shown to exist. Crossreactivity between *Yersinia*, *Francisella tularensis*, *Vibrio*, and a thyroid-stimulating immunoglobulin may also exist. Therefore, results should be interpreted with caution and correlated with clinical information [58].

A polymerase chain reaction from patients suspected of TI is currently suggested, as culture methods for the diagnosis of Y. infection are gradually replaced by molecular tests. DNA microarray for pathogenic organisms, a comparatively new technique that is used to identify multiple genes from different kinds of pathogens, has been used in the diagnosis of Y. infection.

Endoscopy is very useful to detect lesions of the terminal ileum mucosa and obtain biopsies in order to evaluate the degree and kind of inflammation. Findings may vary and are, in most cases, relatively nonspecific. Typically, in patients with YE infection, aphthoid ulcers can be found in the caecum, while in the terminal ileum, small, round elevations and ulcers could be demonstrated. Exudates may be present. The left side of the colon is typically unaffected, but case reports have described left-sided colitis with serotype O:8. In a relevant study involving eight patients with isolation of *Yersinia* in the faeces and serum antibodies against *Yersinia*, the terminal ileum was affected in all, followed by the involvement of the ileocecal valve and the caecum and, in a lesser degree, the ascending colon [59]. The main endoscopic findings were round or oval elevations with or without ulcers in the terminal ileum. However, small ulcers were also detected in the ileocaecal valve as well as in the caecum [59]. It is of interest that these endoscopic findings were seen after five weeks following the symptoms' onset.

The histological findings of *Yersinia* infection are not pathognomonic and usually are only indicative of acute and/or chronic inflammation. Patchy villous atrophy and crypt hyperplasia, with mixed acute and chronic inflammation and focal neutrophilic cryptitis, along with epithelial cell granulomas composed of histiocytes and small T-lymphocytes and plasma monocytes with suppuration of the centres, have been reported [52]. Furthermore, the excess tropism for lymphatic tissue shown by *Yersinia* along with the characteristic histopathological findings in mesenteric lymph nodes suggests that the stimulation of T cells plays a central role in the pathogenesis of this infection [60].

## 6. Treatment Strategies

The treatment of acute severe Yersiniosis includes supportive care with fluid, electrolyte, and nutritional restoration. Admission to the hospital and antibiotic administration should be considered in the elderly people; immunocompromised patients; patients with diabetes, iron overload, and alcoholism; patients on chronic hemodialysis, or on deferoxamine therapy; and patients who are chronically ill. In case of an abdominal abscess, it is necessary to drain it, preferably by surgery. Moreover, surgical exploration is warranted if there is a real possibility of appendicitis, as pseudoappendicitis and appendicitis cannot always be differentiated on a clinical and/or imaging basis. If the appendix is normal during the surgical exploration, then it is crucial to remove lymph nodes for histology.

Drugs that are currently used include quinolones, aminoglycosides, trimethoprim-sulfamethoxazole combination, tetracyclines, and third-generation cephalosporins. In a study with *Yersinia*-triggered reactive arthritis, patients were randomly allocated to receive ciprofloxacin 500 mg twice daily orally or placebo for three months. Faster remission and relief of pain was noticed in the group of patients receiving ciprofloxacin as compared to the control group. Moreover, YE disappeared from the gut-associated lymphoid tissue in all patients receiving ciprofloxacin compared with none of the patients receiving placebo. It was of interest that patients receiving placebo had more and prolonged circulating IgA antibodies against YOPs than patients treated with ciprofloxacin [61]. Other studies have shown that patients with *Yersinia*-triggered arthritis show persistent serum IgA antibodies against YOPs as compared with patients having uncomplicated Y. infection. It is possible that prolonged administration of antibiotics could eliminate persistent bacilli from the gut, thus eliminating the circulating IgA antibodies from the serum [62]. In patients treated successfully with ciprofloxacin only, IgG antibodies against YopD can be found. Patients receiving ciprofloxacin rarely develop antibodies against YopH, and LSO rarely develops antibodies against YOPM.

In conclusion, ciprofloxacin influences the antibody production by eliminating the trigger, thus preventing the development of a chronic situation. Preventive measures include hand washing and meticulous food processing. The outcome of the infection is generally favorable [63].

## 7. Links between *Yersinia* Infection and Crohn's Disease

There is some evidence to suggest that YE infection may increase the risk of subsequent development of IBD. An association between YE infection and ulcerative colitis was suggested, and a connection with CD also exists [52]. For example, there have been reports of IBD diagnosed at laparotomy in individuals in whom *Yersinia* was isolated from faecal samples or who had positive serum tests [53]. Furthermore, YE DNA has been detected in the histology of colonic resections and mesenteric lymph nodes in a series of CD patients [64]. A case control study showed that the incidence of IBD was higher in patients with positive YE serology than in the antibody negative group [53]. However, the incidence of concurrent infections in patients who have a relapse of IBD is reported to vary widely and may well be influenced by the enthusiasm with which the identification of an infective source is investigated [65].

A large number of epidemiological studies originating from the developed world suggest that IBD is linked to the modern western way of life. This assumption is further supported by epidemiological data originating from the developing countries in North Africa, South Asia, or South America showing a rising incidence of IBD following the improvement in the standards of living in these countries. There is no doubt that the economic development, leading to improved hygiene and other changes in lifestyle may play a role in the increase in the incidence of IBD. Refrigeration, an element of the so-called “cold hypothesis,” possibly represents a link between the modern way of life, bacteria, diet, and domestic hygiene.

The “cold chain hypothesis” provides a logical platform concerning the aetiology of IBD by linking dietary factors, microbial agents present in the digestive tract, and the possible association between home comfort and IBD. This hypothesis suggests that psychrotrophic bacteria, such as *Yersinia* spp., contribute to the development of CD. It has been postulated that CD is a result of a defect in host recognition by pathogenic bacteria components that usually escape the immune response (e.g., YOP molecules), which results in an excessive host response to these bacteria [66]. On the other hand, because of the existence of defects in mucosal barrier function and immunological function, patients with CD may have increased susceptibility to infection by *Yersinia* spp. [49].

In favor of the “cold hypothesis” is the study by Forbes and Kalantzis who found that the average age of getting a fridge was more than 4 years earlier in older IBD patients than in age-matched controls [67]. It is possible that refrigeration is only a confounding factor associated with CD rather than an element of the causative chain leading to CD [66]. The potential link between the refrigeration of food and CD is possibly mediated via the exposure to YE which has the ability to survive or develop even at low temperatures (−1°C and +10°C) [68]. Adaptation to cold involves changes in protein synthesis and in cell membranes to overcome diminished transcriptional and translational efficiency and reduced fluidity of cell membranes.

For many years, it was thought that *Yersinia* infection can be seen mainly in the Northern countries of Europe being extremely rare in countries of the Southern Europe, including Greece. However, during the last years, we have observed in our IBD centre the presence of IgG and IgA antibodies against a panel of seven YOP antigens of YE (western blot analysis) in a large cohort of patients with CD (unpublished data). A large proportion of our patients with either active or inactive CD had positive antibodies indicating a coexistence of *Yersinia* infection in some patients with CD.

Ciprofloxacin represents an active antibiotic in patients with CD being concurrently a first-line antibiotic against YE. The benefits derived from the administration of ciprofloxacin could be related (among others) to its effect against Yersinia.

YE and YP often cause self-limited enteritis and mesenteric lymphadenitis in children. In histological specimens of both gut and lymph nodes, the formation of granulomas can be seen. As it was mentioned before, *Yersinia* can produce urease which is essential in gastritis, a finding which could explain the high frequency of focally enhanced gastritis observed in CD [69]. *Yersinia* is also able to produce a heat-shock protein which has been considered as a cause of diarrhea. *Yersinia* spp. may also induce reactive arthritis and erythema nodosum, two clinical situations that could be found in many patients with CD.

It could be argued that chronic exposure to *Yersinia* spp. may contribute to maintain an intestinal inflammation. As it was previously emphasized, *Yersinia* spp. are able to produce an invasin which facilitates cell penetration by bacteria [70]. *Yersinia* infection in mice shows histological alterations of vesicles centred on lymphoid follicles which finally can take the form of a microabscess [71]. Several authors have reported that aphthoid ulcerations are centred on lymphoid follicles [72].

*Yersinia pseudotuberculosis* can cause NF-*κ*B activation by targeting the signal transduction pathways [73]. It is well known that the NOD2 gene represents an activator of the NF-*κ*B pathway and that the relationship between the microbial lipopolysaccharide and CD is due to mutations in NOD2 that predispose to the development of CD [74]. A frameshift mutation in NOD2 results in innate hyporesponsiveness to some bacterial components, thus facilitating an abnormal adaptive response to enteric microbes [75]. The recent development of domestic refrigeration—with a consequent higher exposure to low doses of *Yersinia* spp. in food products—might explain the outbreak of CD in developed countries. It remains to be seen in large studies if *Yersinia* may be present in CD lesions, as well as to define the virulence factors and confirm that CD is associated with an immune host response towards these bacteria.

## 8. Conclusion

Yersiniosis is an acute diarrheal illness caused by 3 species of YE, YP, and *Y. pestis*. Diagnosis is based on a positive stool culture and the detection of serum YOP antigens using the western blot analysis. The initial treatment for uncomplicated diarrhea involves hydration, nutrition, and electrolyte replacement. Emergency care should be sought if the patient develops severe symptoms or complications. Hand washing and the avoidance of raw pork products are the best way to prevent these infections. Enteropathogenic YE and YP are of significant concern to the pork industry. The ability of the *Yersiniae* to replicate and thrive at refrigeration temperatures suggests that future surveillance measures are inevitable. At present, enteropathogenic *Yersinia* cases are likely underestimated. We strongly believe that Yersiniosis should be included in the diagnostic work-up in all patients with TI and that specialists should be cautious with establishing the diagnosis of IBD in such patients. Thorough surveillance measures will allow a more precise estimation of national Yersiniosis trends and help us understand which measures should be taken in order to reduce the incidence of *Yersinia* infection in the general population.

## Additional Points

*Core Tip*. Inflammation in the terminal ileum might be—among others—due to the infection by *Yersinia*, in conjunction or not with CD. Despite the fact that differences in endoscopy and histology between Yersiniosis and CD certainly exist, the exact diagnosis is not always easy. Apart from performing a thorough diagnostic work-up in every patient with terminal ileitis, the estimation of serum antibodies against YOP antigens (western blot) needs to be done as terminal ileitis is not always CD.
